# Linking human behaviours and malaria vector biting risk in south-eastern Tanzania

**DOI:** 10.1371/journal.pone.0217414

**Published:** 2019-06-03

**Authors:** Marceline F. Finda, Irene R. Moshi, April Monroe, Alex J. Limwagu, Anna P. Nyoni, Johnson K. Swai, Halfan S. Ngowo, Elihaika G. Minja, Lea P. Toe, Emmanuel W. Kaindoa, Maureen Coetzee, Lenore Manderson, Fredros O. Okumu

**Affiliations:** 1 Environmental Health and Ecological Science Department, Ifakara Health Institute, Ifakara, Tanzania; 2 School of Public Health, Faculty of Health Sciences, University of the Witwatersrand, South Africa; 3 Johns Hopkins Center for Communication Programs, Baltimore, MD, United States of America; 4 University of Basel, Basel, Switzerland; 5 Swiss Tropical and Public Health Institute (Swiss TPH), Basel, Switzerland; 6 Institut de Recherche en Sciences de la Santé, Bobo-Dioulasso, Burkina Faso; 7 Wits Research Institute for Malaria, School of Pathology, Faculty of Health Sciences, University of the Witwatersrand, Braamofontein, South Africa; 8 Institute of Biodiversity, Animal Health and Comparative Medicine, University of Glasgow; Instituto Rene Rachou, BRAZIL

## Abstract

To accelerate malaria elimination in areas where core interventions such as insecticide-treated nets (ITNs) are already widely used, it is crucial to consider additional factors associated with persistent transmission. Qualitative data on human behaviours and perceptions regarding malaria risk was triangulated with quantitative data on *Anopheles* mosquito bites occurring indoors and outdoors in south-eastern Tanzania communities where ITNS are already used but lower level malaria transmission persists. Each night (18:00h-07:00h), trained residents recorded human activities indoors, in peri-domestic outdoor areas, and in communal gatherings. Host-seeking mosquitoes were repeatedly collected indoors and outdoors hourly, using miniaturized exposure-free double net traps (DN-Mini) occupied by volunteers. In-depth interviews were conducted with household representatives to explore perceptions on persistent malaria and its control. Higher proportions of people stayed outdoors than indoors in early-evening and early-morning hours, resulting in higher exposures outdoors than indoors during these times. However, exposure during late-night hours (22:00h–05:00h) occurred mostly indoors. Some of the popular activities that kept people outdoors included cooking, eating, relaxing and playing. All households had at least one bed net, and 83.9% of people had access to ITNs. Average ITN use was 96.3%, preventing most indoor exposure. Participants recorgnized the importance of ITNs but also noted that the nets were not perfect. No complementary interventions were reported being used widely. Most people believed transmission happens after midnight. We conclude that insecticide-treated nets, where properly used, can still prevent most indoor exposures, but significant risk continues unabated before bedtime, outdoors and at communal gatherings. Such exposure is greatest for rural and low-income households. There is therefore an urgent need for complementary interventions, particularly those targeting outdoor-biting and are applicable for all people including the marginalised populations such as migratory farmers and fishermen. Besides, the differences in community understanding of ongoing transmission, and feedback on imperfections of ITNs should be considered when updating malaria-related communication and interventions.

## Background

Malaria transmission in rural south-eastern Tanzania has significantly decreased over the past three decades [1,2], although the area is still classified as meso- or hyper-endemic based on the most recent surveys of malaria prevalence in school children [3]. Various factors have contributed to these declines, including improved case management and effective vector control measures, particularly wide-spread use of insecticide-treated nets (ITNs) [4–6]. Other factors such as urbanization and improved livelihood may have also played a role [7], although the exact measure of their contribution is not known. Factors associated with the persisting transmission include resistance to pyrethroids commonly used on insecticide-treated nets (the most common vector control intervention) [8], increased proportion of malaria mosquitoes biting people outdoors [8–10] and occupational factors such as migratory farming activities [11]. Indeed, interventions like ITNs offer protection mainly when people are sleeping indoors, leaving them mostly vulnerable outdoors [9,12,13]. To deploy effective complimentary interventions and to accelerate elimination efforts, it is therefore crucial to also investigate these additional factors and to identify potential opportunities for improvement.

Social, cultural and economic factors such as customs, education, income levels, environment, lifestyles and values are important determinants of malaria transmission [14]. They can influence access to and use of bed nets, health care seeking behaviours and adherence to interventions[14]. In recent years, there has been growing evidence on correlations between outdoor-biting and malaria transmission [10,13]. However, since mosquitoes only bite people where they overlap in space and time [15–17], human activities are equally vital drivers of persistent transmission. Moreover, community perceptions of risk, burden and severity of malaria may influence control [18].

The aim of this study was to identify key anthropological and entomological drivers of persistent malaria transmission in the Kilombero valley in south-eastern Tanzania, an area where ITNs are widely used but malaria transmission persists. It investigated correlations between human behaviours and mosquito biting risk indoors and outdoors in rural and urban Tanzanian villages. The term persistent malaria transmission is used refer to the transmission that continues despite wide coverage of the main vector control tool in the area, i.e. ITNs.

## Methods

### Ethical considerations

Meetings with local community leaders and community members in the study sites were held and the aim and procedures of the study were explained. Separate written consent was obtained from heads of households for observations, mosquito collections and photos. Participants of in-depth interviews also provided written informed consents. Additionally, consent was obtained from owners of the non-peridomestic places observed. Ethical approval for the study was obtained from the WHO ethical review committee (Protocol ID: ERC: 0002672), Ifakara Health Institute Institutional Review Board (IHI/IRB/No: 35–2015) and from the Medical Research Coordinating Committee (MRCC) at the National Institutes of Medical Research (NIMR), Ref: NIMR/HQ/R.8a/Vol.IX/2162). Ethical approval was also obtained from the Faculty of Health Sciences Ethical Review Board at the University of the Witwatersrand with an Approval Certificate No: R149/49. Permission to publish this study was obtained from NIMR, ref: NIMR/HQ/P.12 VOL XXVI/34.

### Study area and period

**Fig 1:**
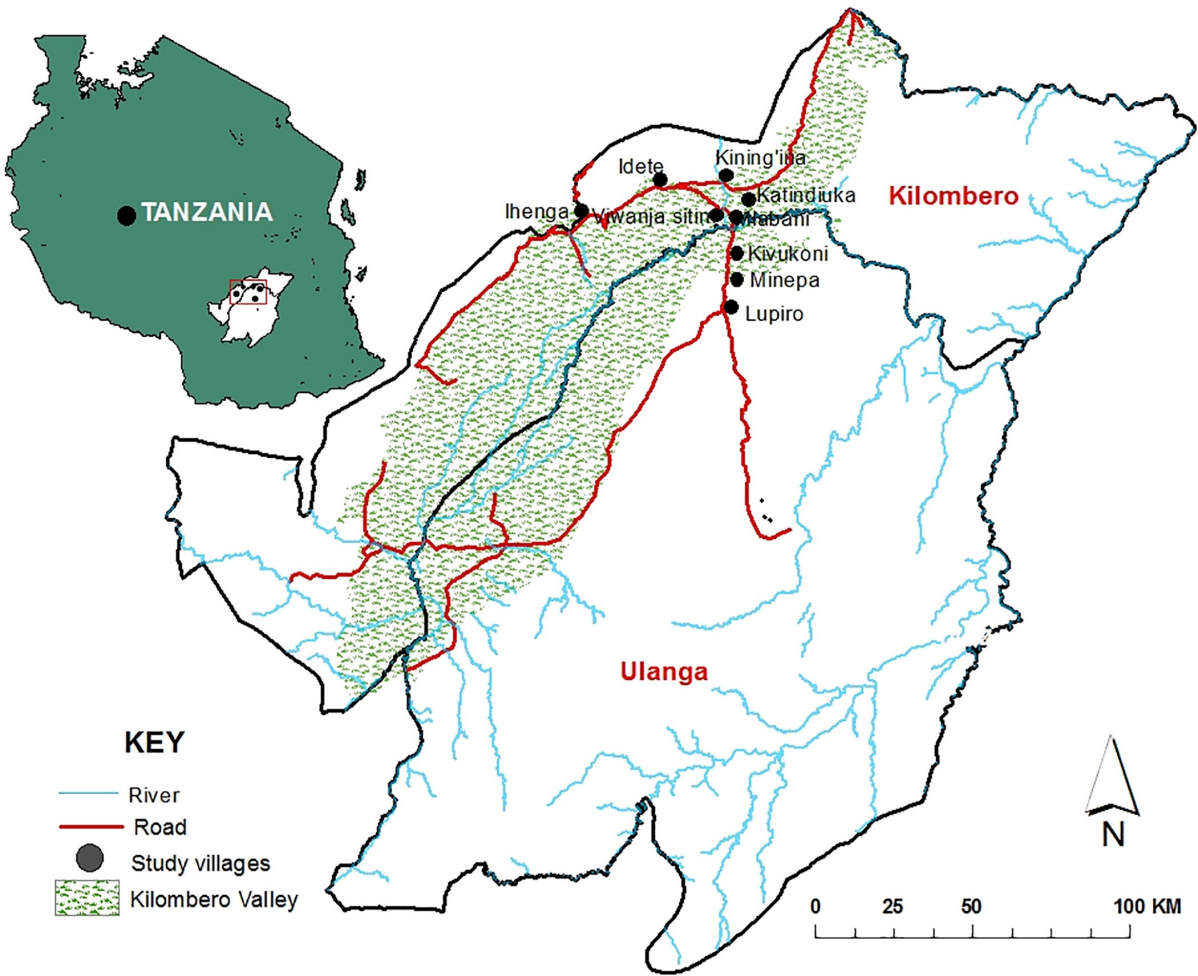
Map of the study areas showing the nine villages of the Kilombero valley where the entomological and human activity surveys were conducted.

This study was conducted between August 2016 and June 2017 in nine villages in Ulanga and Kilombero districts, both in the Kilombero valley in south-eastern Tanzania (Fig 1). In Ulanga district, it was done in the rural Kivukoni, Minepa and Lupiro villages, where inhabitants are predominantly rice farmers. In Kilombero district however, study sites included urban and peri-urban sites of Katindiuka, Ifakara Mjini and Viwanja Sitini, where inhabitants are small business owners or have formal employment, but frequently cultivate farms in the distant river valley.

Additional surveys were done in rural Kilombero district in Idete, Ihenga and Kining’ina villages, occupied by farmers and pastoralists. Mosquito densities are high most of the year, peaking between March and May [19,20]. Malaria transmission is primarily mediated by *Anopheles arabiensis* and *Anopheles funestus*, the latter contributing >80% [19]. Entomological surveys show that transmission dropped from 319 infectious bites per person per year (ib/yr) in the 1990s [21] to 18 ib/yr in the rural settings in 2016 [19], even though actual parasite prevalence is still significant [3]. In the urban settings (Ifakara town and the surrounding peri-urban wards), the declines in transmission were even greater, from 30 ib/yr to less than 1 ib/yr between 2000 and 2016 [22,23].

### Household selection

Ninety households were randomly selected, from lists obtained from Ifakara Health and Demographic Surveillance System (HDSS) [24], across nine villages in Ulanga and Kilombero districts. With help from community leaders, the study team visited the selected households and recruited them upon informed consent. Human activities and behaviours were monitored in all 90 households, after which complementary entomological and qualitative surveys were conducted in a subset of households as detailed below. All data collection was done for three months in dry season (August–November 2016 & July–October 2017) and three months in wet season (March—June 2017).

### Study design

**Fig 2:**
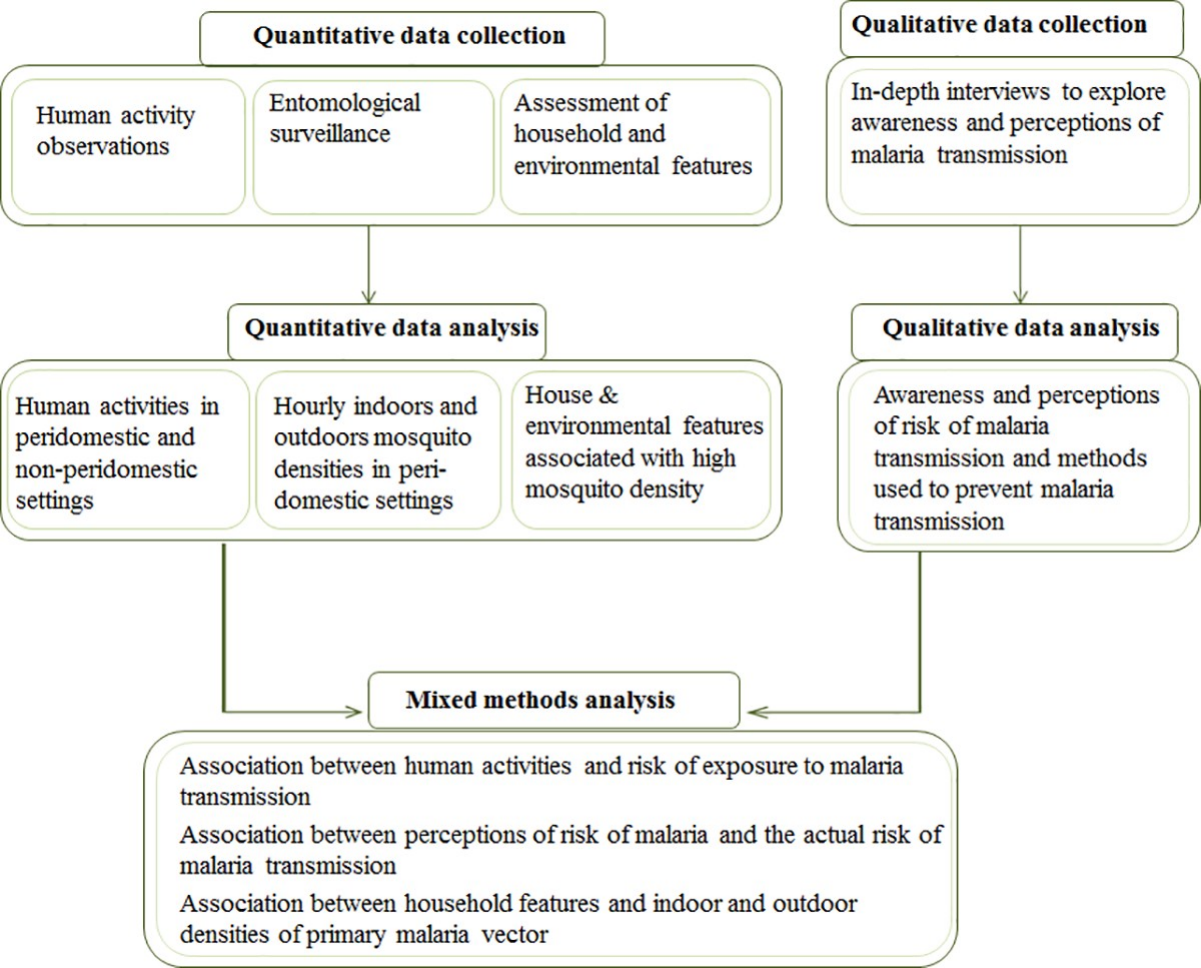
Illustration of the triangulation mixed methods design used to explore and assess factors associated with mosquito biting exposure in Ulanga and Kilombero districts, south-eastern Tanzania.

A concurrent triangulation mixed methods design [25] was used to explore and assess factors associated with persistent malaria transmission. The quantitative component (human activity observations and entomological surveillance) and qualitative component (in-depth interviews) were done concurently (Fig 2). For purposes of this study, peri-domestic spaces were defined as areas surrounding the observed house, limited to a 10-meter radius. Non-peri-domestic settings were settings where people tended to gather away from the observed houses, e.g. other homes in the community, bars, movie kiosks and cultural or religious gatherings (e.g. weddings and prayer events). The in-depth interviews explored people’s perceptions on malaria transmission risk. Additionally, household and surrounding environments were observed and characterised to examine features potentially linked to mosquito biting.

#### Monitoring indoor and outdoor activities in the peri-domestic areas

Observations of human locations and activities in the peri-domestic areas were conducted in all the 90 households (ten per village). Consenting adult household members or relatives were recruited and provided with three-day training on how to fill the observation forms for household activities (S1 File). The observers recorded the number of people doing the activities at different times of night, every half hour from 18:00h to 07:00h. Only household members were observed. During each time period, the number of household members in the different locations (indoors, outdoors and away) was recorded. For household members that were indoors or in peridomestic outdoor areas, their activities were recorded from a set list of pre-defined activities. Note that the list of activities captured net usage. The number of household members that were away from the house was calculated from the total number household members that were indoors and outdoors subtracted from the overall total number of household members. Each observed household member was classified by sex and age (i.e. adults and children of school going age (6-years and above) and children below school-going age (under 6s)). The observations were done for three days every month, for three months in rainy season and three months in dry season. Observations were done every other day to allow for a day of entomological data collections in between.

#### Monitoring activities in non peri-domestic places

Additional observations were done in non-peri-domestic places where people tended to gather in evenings and night hours. These included bars and kiosks where people watch movies or football games, and events such as weddings, funerals and other celebrations. Heads of household and community leaders were asked to inform the research team of any night-time gatherings in their communities, after which trained community members conducted the observations. Only places or events whose owners had consented were observed. Observations were done on an hourly basis, starting from 18:00h to 07:00h the following morning, similar to the peri-domestic observations. A summary of these activities is presented in supplement file (S5 File).

#### Entomological collections

In the initial surveys, CDC Light Traps [26] were used to assess indoor and outdoor mosquito densities. Special bags were made to hold trapped mosquitoes for each hour, from 18:00h to 07:00h. The traps were set near volunteer-occupied untreated nets [27]. Each household was provided with two new mosquito nets; one for outdoor and another for indoor sampling. Outdoors the bed net was tied on a pole or tree between 5 and 10 meters from the selected house, and an adult male volunteer sat on a chair under the bed net. The volunteer was responsible for exchanging the CDC light trap bags indoors and outdoors. This activity was done in five households randomly selected from the ten households that were being observed in each of the nine villages, totalling to 45 households. The collections were done every other day for three days every month, for three months in wet and three in dry season. The collected mosquitoes identified morphologically [28] then sorted by taxa and sex.

**Fig 3:**
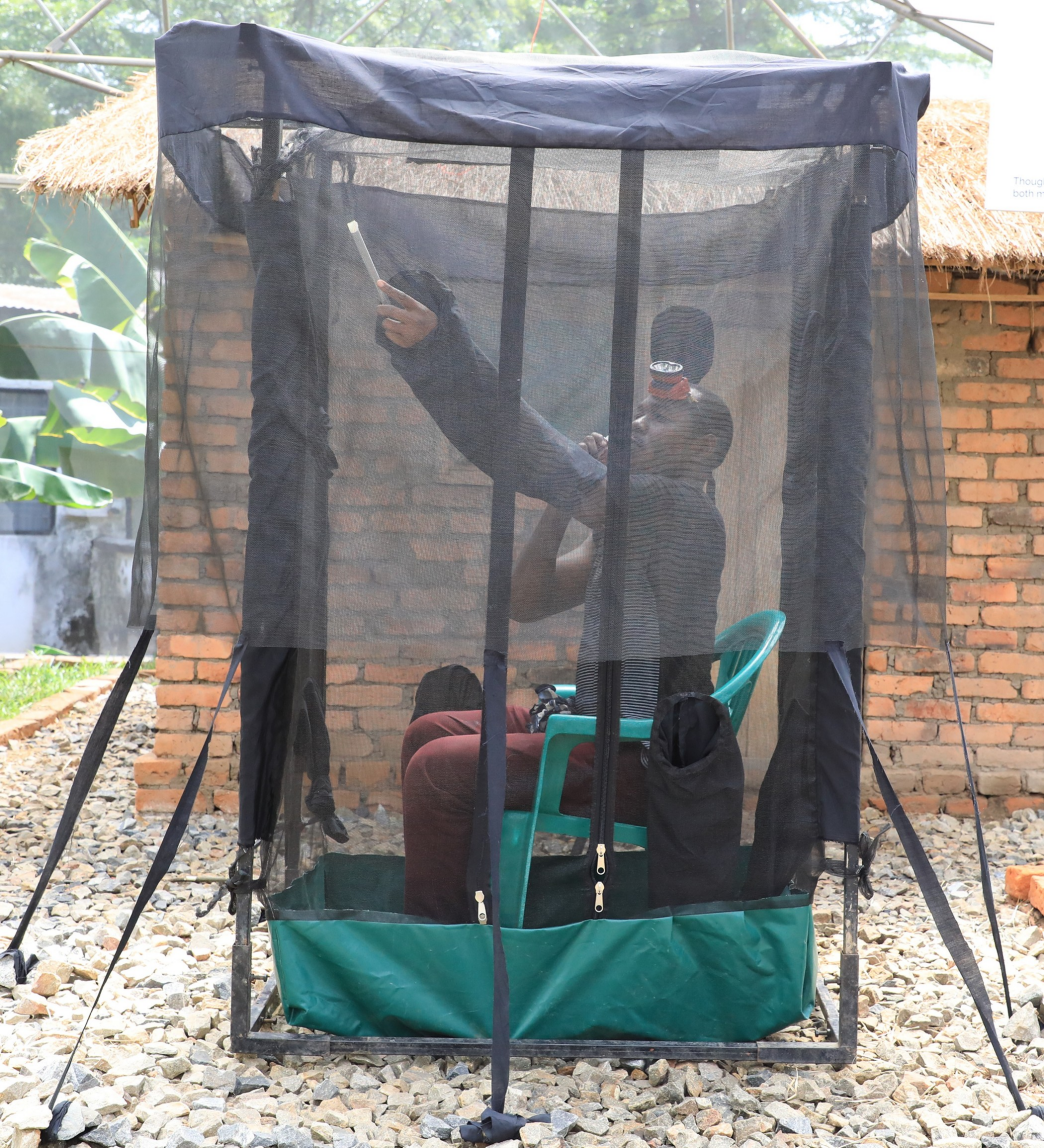
Miniaturized double-net trap (DN-Mini) for comparison of indoor and outdoor mosquito densities.

These initial tests revealed that CDC-light traps were not effective for outdoor-indoor comparisons of biting risks, as the data did not match known behaviors of *Anopheles* in the area [20]. Therefore, additional mosquito collections were done using a miniaturized double net trap (DN-Mini). DN-Mini is an adaptation of the bed-net trap previously described in the WHO 1975 practical entomology manual [29], that was later on adapted by Tangena et al [30]. DN-Mini enables exposure-free trapping and comparison of biting mosquitoes indoors and outdoors (Limwagu et al, unpublished). It is made with UV-resistant fibre glass over four metal poles and canvas base (Fig 3). This trap has also recently been used to assess efficacy of repellent-treated eave space ribbons [30]. The trap has dimensions of 60cm width, 100cm length and 180cm height, and has an outer overhang netting (100cm-height) to allow entry of mosquitoes between the layers, while preventing escape. The inner netting has multiple sleeves through which a volunteer can retrieve the trapped mosquitoes. Two DN-Mini traps were placed at each house, one indoors and another outdoors, with adult male volunteers sitting inside each, as bait and collecting the host-mosquitoes caught between the double layers. These exposure-free DN-Mini collections were done hourly between 18:00h and 07:00h. For every hour of collection, the volunteers sat inside the DN-Mini for 45 minutes and spent 15 minutes retrieving mosquitoes trapped between the double layers. These DN-Mini collections were done at eight houses in four of the nine villages (Kining’ina, Kivukoni, Minepa and Lupiro) for 20 nights at each house.

#### Assessment of household and environmental features

Each of the 90 houses and their surrounding environments were characterized. Features observed included materials used for roofs and walls, number of windows and doors, types of window and door covers, number and condition of bed nets, distance to water source, types of animals kept and places animals are kept within the household at night. These observations were done using closed-ended questionnaire and direct observations.

#### Assessment of knowledge, attitude, and perceptions of malaria transmission

In-depth interviews were conducted with household members. The interviews were audio-recorded, transcribed and translated immediately, incorporating associated field notes. Discussions focused on participants’ understanding of malaria transmission and prevention, their perceptions of risk, methods used for protection, and perceptions of effectiveness of these methods. Each interview lasted 30 to 60 minutes and was conducted either at the participants’ home or place of work.

### Processing and analysis of quantitative data

*Observations and entomological data*: Peri-domestic and non-peri-domestic activity data were entered in Microsoft Excel spreadsheets after which proportions of people indoors, outdoors and away doing different activities hourly was calculated and presented using bar graphs, tables and pie charts. Entomological data was recorded in standardized data forms used at Ifakara Health Institute. Proportions biting indoors and outdoors were assessed for each mosquito species. Nightly mosquito biting patterns of the main malaria vector species, *An*. *arabiensis* and *An*. *funestus* were computed by calculating hourly catches indoors and outdoors.

Association between household characteristics and mosquito density was analysed using generalized linear model following a negative binomial approach. Mosquito densities were modelled as a response variable while household characteristics (electricity, location of the houses, window structure) were included in the model as predictor variable. Each mosquito species were modelled separately. Relative risk (RR) and their respective 95% Confidence intervals were reported. The results were considered significance when p-value was less than 0.05.

*Human exposure to mosquito bites*: Data collected using DN-Mini was used for comparing indoor and outdoor exposures to malaria vector bites. The vector-host behavioural interaction was assessed by first estimating proportion of human exposure to mosquito bites indoors and outdoors. This was done by weighting mean indoor and outdoor-biting densities of both *An*. *arabiensis* and *An*. *funestus* for each hour respective to proportions of humans observed indoors and outdoors at that hour[31,32]. Percentage protection offered by ITNs was calculated assuming maximum protection efficacy of 73.3% of ITNs, based on previous studies in Tanzania[31,33]. Risk of exposure was also compared between the households with and without window screens as well as in households with and without electricity.

*Net ownership*, *access and use*: Household net ownership was estimated by calculating proportion of households owning at least one bed net. Net access was estimated as proportion of the household members who could be covered by available nets, assuming that each ITN is used by two people within the household [34]. Net use was calculated as a proportion of people sleeping or resting under the bed nets, as established during the household observations.

### Processing and analysis of qualitative data

*In-depth interviews*: The transcripts were reviewed and imported into Nvivo 12 Plus software [35], after which deductive and inductive coding was used to extract emergent themes [36]; objectives of the study and interview guides were used to develop deductive or topic codes. Other codes were generated inductively based on content of the transcripts. Similar codes were grouped and emergent patterns used to identify themes. The extracted themes included participant's views and perceptions on: a) how malaria is transmitted from one person to another, b) when malaria mosquitoes were deemed active, c) changes in malaria transmission over the years, d) actual risk of getting malaria, e) malaria prevention methods, and f) challenges faced in protecting their families. Quotes from the participants are provided as support for the themes.

## Results

### Household and environmental features

The majority of houses in rural and urban settings had brick walls and iron-sheet roofs (Table 1). It was common to observe multiple family units living as one household, especially in the urban settings. Average number of family units per household was 1.3 (range:1–6) units/ household, while average family size was 5.3 people and was higher in rural than urban settings (Table 1). Bed net ownership (proportion of households with at least one net) was 100%, with an average of 3.7 nets per household (range: 1–8 nets). Bed net access was 83.9%, and the proportion of net use among the household members was 96.3% (98.2% among female and 94.4% among males). A majority (81%) of the bed nets in the rural settings were still intact (zero holes), as the observation was just after the universal bed net distribution in 2017. However, urban observations were in May and June 2016 before the universal net distribution, and only one third of the nets were intact (Table 1).

**Table 1:** Characteristics of the study participants and their houses in the nine study villages in Ulanga and Kilombero districts, south-eastern Tanzania.

Characteristic		All sites	Urban sites**	Rural sites
Number of households		90	30	60
Average number of people per household		7.9	7.1	8.4
Average number of family units per household		1.5	1.7	1.3
Average number of people per family		5.3	4.2	6.5
Gender composition in households	Male	50.9% (n = 374)	46.0% (n = 104)	53.0% (n = 270)
	Female	49.1% (n = 361)	54.0% (n = 122)	47.0% (n = 239)
Age distribution	5 and under	15.5% (n = 114)	10.2% (n = 23)	17.9% (n = 91)
	6–17	32.1% (n = 236)	41.2% (n = 93)	28.1% (n = 143)
	18–45	41.8% (n = 307)	33.1% (n = 75)	45.6% (n = 232)
	>46	10.6% (n = 78)	15.5% (n = 35)	8.4% (n = 43)
*Characteristics of the houses*				
Main wall material	Bricks & cement	78.0% (n = 70)	90.0% (n = 27)	71.6% (n = 43)
	Mud & sticks	22.0% (n = 20)	10.0% (n = 3)	28.4% (n = 17)
Main roof material	Metal (iron-sheets)	74.4% (n = 67)	86.7% (n = 26)	66.7% (n = 40)
	Thatched	25.6% (n = 23)	13.3% (n = 4)	33.3% (n = 20)
Window covers	Netting screen	52.2% (n = 47)	63.3% (n = 19)	41.7% (n = 25)
	Unscreened	47.8% (n = 43)	36.7% (n = 11)	58.3% (n = 35)
Outlet door shutters/covers	Wood/metal	94.4% (n = 85)	93.3% (n = 28)	93.3% (n = 56)
	Other covers	4.4% (n = 4)	6.7 (n = 2)	5.0% (n = 3)
	No cover	1.1% (n = 1)	0.0% (n = 0)	1.7% (n = 1)
*Bed net access and usage*				
Average number of sleeping places/household		3.5	3.4	3.6
Average number of sleeping places with nets		3.5	3.3	3.5
Average number of bed nets used on the day of observation		3.3	3.2	3.4
Condition of the bed nets	Intact	63.7%	31.4%	81.9%
	Repaired	18.3%	40.2%	6.0%
	1–3 holes	11.6%	12.7%	11.0%
	4–6 holes	3.9%	10.8%	0.0%
	7+ holes	2.5%	4.9%	1.1%

**Urban sites in this case refer to Ifakara town and the surrounding peri-urban wards

### Proportions of people indoors, outdoors or away from home at different times of night

**Fig 4:**
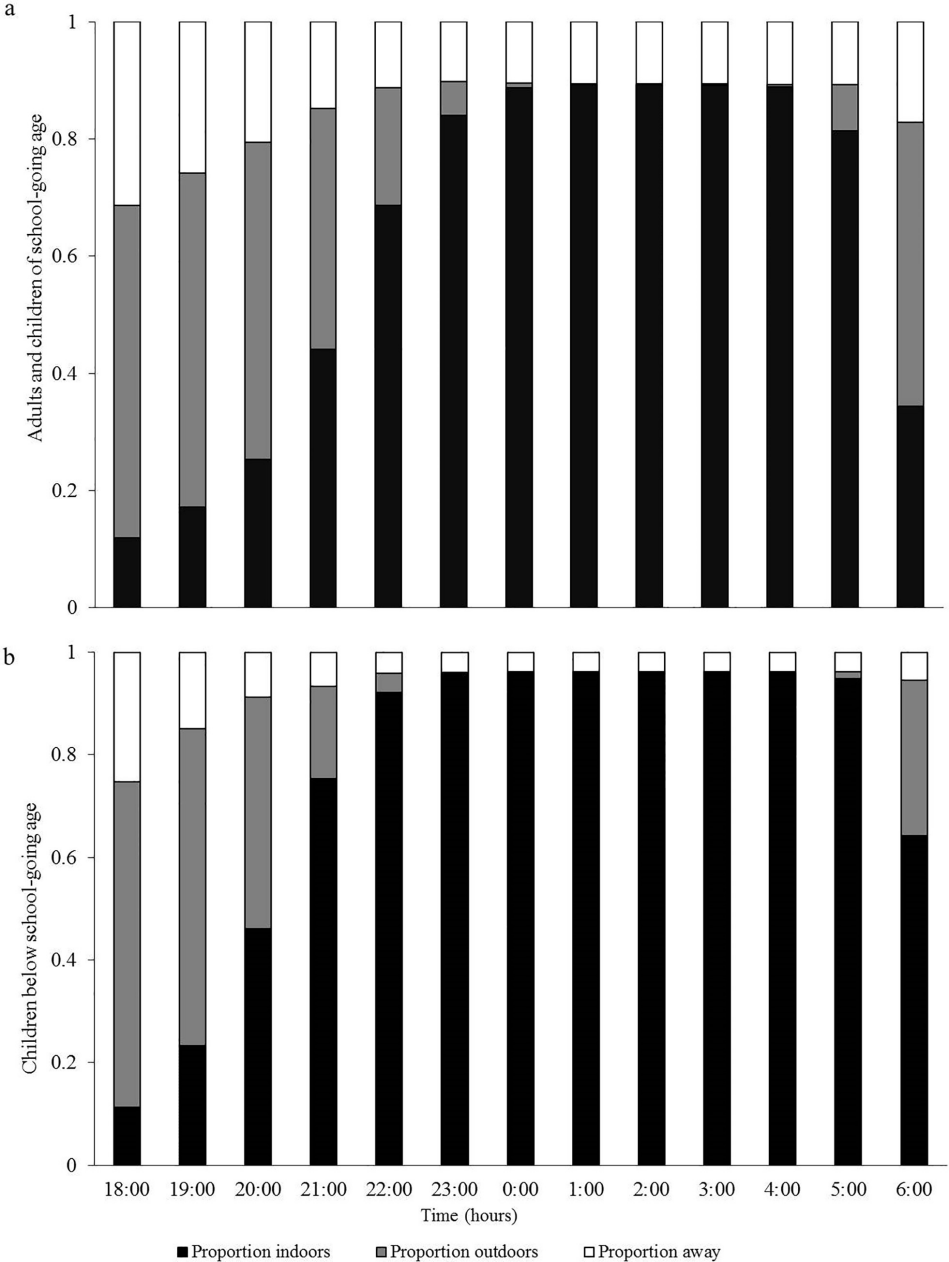
**Proportion of household members indoors, outdoors or away from home at different times of night: (a) Adults and children of school-going age, i.e. all household members six years or older; (b) Children below school-going age, i.e. all members <6yrs**.

Proportions of people indoors, outdoors or away from their homes are shown in Fig 4. In both rural and urban settings, a majority of the household members were either outdoors or away between 18:00h and 21:00h, and mostly indoors between 22:00h and 05:00h, after which time the outdoor proportion increased. Children below school age went indoors earlier than other household members; more than 75% and 90% of the children were indoors by 21:00h and 22:00h respectively, compared to 44.1% and 68.6% of other household members indoors by 21:00h and 22:00h.

### Common activities in the peri-domestic space

**Fig 5:**
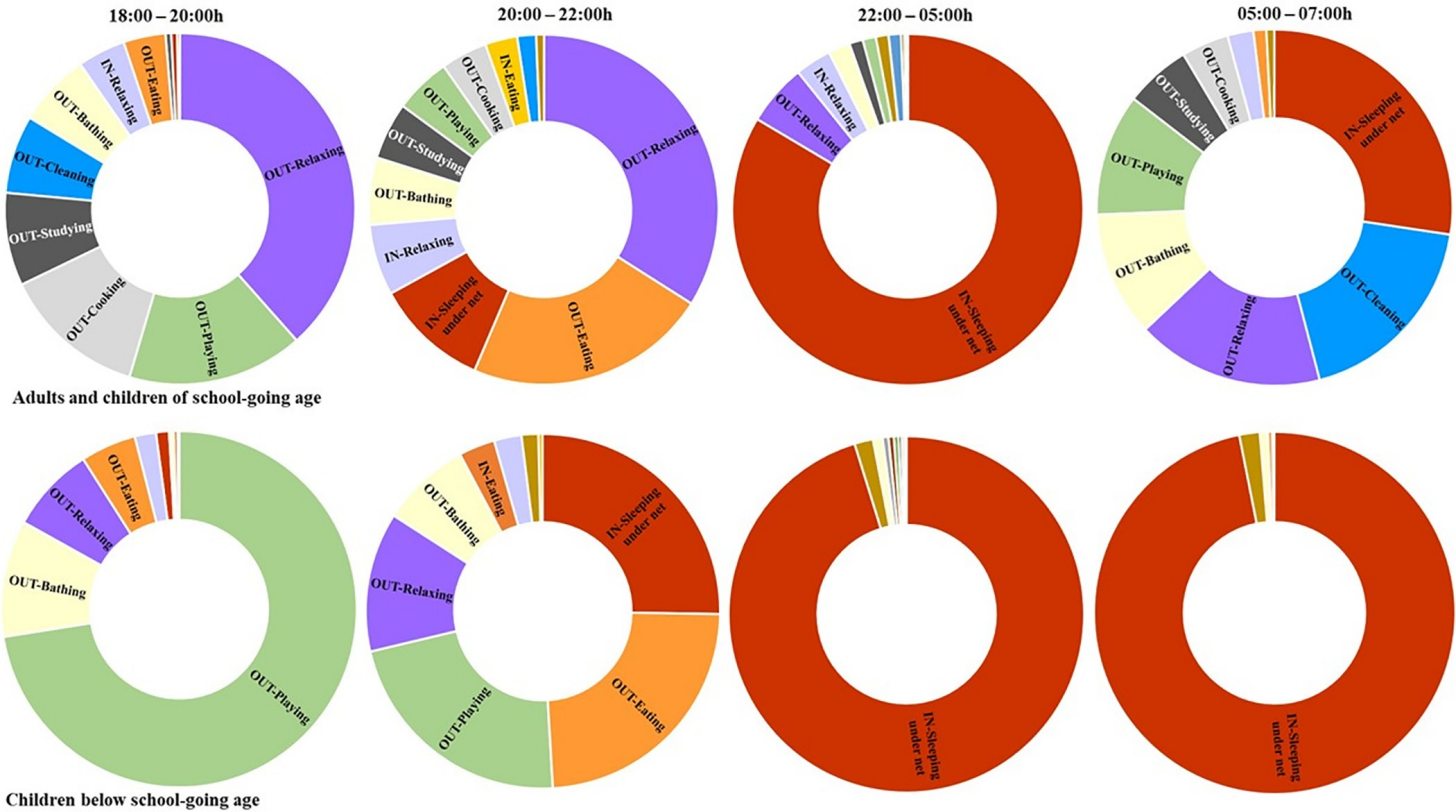
**Indoor and outdoor activities observed among household members in the peri-domestic areas: a) Adults and children of school-going age, i.e. all household members of six years and above; (b) Children below school-going age, i.e. all household members of below six years**.

In early evenings (between 18:00h and 20:00h), most children < 6 years (below school age) were playing outside, and others were being bathed, eating or relaxing (i.e. resting, chatting, listening to radio or watching TV) (Fig 5). Adults and children over 6 years were also mostly outdoors from 18:00 to 20:00h, mainly relaxing, cooking, playing, reading or studying and cleaning (i.e. sweeping yards, washing dishes or doing laundry) (Fig 5). Indoor activities, mostly sleeping under bed nets, peaked after 20:00h until 05:00h, after which time outdoor activities started picking up.

There was a clear gender difference in the popular activities observed indoors and outdoors. Activities like cooking, cleaning, eating and sleeping under bed net were more popular among female household members while relaxing indoors, bathing indoors and studying outdoors were more popular among the male household members. While there were household members sleeping outdoors without bed nets during the early night hours, no household members were observed sleeping under a bed net outdoors (Table 2).

**Table 2:** Gender differences in in the popular peri-domestic activities observed among household members of 6 years and above.

Activities	Indoor			Outdoor		
	N	RR (95% CI)	p-value	N	RR (95% CI)	p-value
*Relaxing*						
Females	88	1		1081	1	
Males	185	2.10 (1.63, 2.71)	p<0.001	1079	0.10 (0.92, 1.09)	p = 0.966
*Cooking*						
Females	53	1		458	1	
Males	3	0.056 (0.02, 0.18)	p<0.001	40	0.09 (0.06, 0.12)	p<0.001
*Playing*						
Females	116	1		453	1	
Males	124	1.07 (0.83, 1.38)	p = 0.606	309	0.68 (0.59, 0.79)	p = 0.01
*Bathing*						
Females	41	1		304	1	
Males	295	7.44 (5.31, 10.45)	p<0.001	295	0.97 (0.82, 1.13)	p = 0.713
*Eating*						
Females	410	1		383	1	
Males	33	0.08 (0.06, 0.11)	p<0.001	299	0.78 (0.67, 0.91)	p = 0.001
*Cleaning*						
Females	94	1		558	1	
Males	32	0.34 (0.22, 0.51)	p<0.001	87	0.16 (0.12, 0.20)	p<0.001
*Studying*						
Females	41	1		135	1	
Males	39	0.95 (0.61, 1.47)	p = 0.822	341	2.53 (2.07, 3.08)	p<0.001
*Sleeping under bed net*						
Females	2653	1		0	*NA*	*NA*
Males	1694	0.64 (0.60, 0.68)	p<0.001	0	*NA*	*NA*
*Sleeping without bed net*						
Females	54	1		172	1	
Males	55	1.02 (0.70, 1.48)	p = 0.924	110	0.64 (0.50, 0.81)	p<0.001

### Activities that keep people away from home

A total of 32 non peri-domestic communal activities were observed in the nine villages. These included funerals, weddings, football matches, baptisms, religious activities (e.g. first communion) and 40^th^-day memorials, a common celebration to mark the end of mourning period after a death in a family. Other activities included gatherings in bars, movie kiosks and other informal settings. Movies, football-kiosks and bars were mostly populated by males (67%), while both males and females participated in all other activities. Activities related to funerals and parties were mostly outdoors, and many lasted all night, while activities associated with bars, movies and football kiosks were both indoors and outdoors, and mostly lasted until midnight. Other places that people were likely to be included fishing camps, or farms (people typically relocated to their distant farms in the river valley during the farming seasons and stayed for up to six months at a time.

### Densities of host-seeking malaria mosquitoes indoors and outdoors

A summary of all mosquito catches is presented in Table 3. A total of 165,028 mosquitoes were caught from the nine villages, of which 94.8% (156,456) were sampled by CDC-light traps and 5.2% (8,572) by DN-Mini trap. *Culex* species comprised 88.3%, *Anopheles*, 8.4%, *Mansonia*, 2.5% and other species 0.8%. Of the *Anopheles* (n = 13,822), *An*. *gambiae s*.*l* comprised of 90.6%, *An*. *funestus*, 3.7% and other *Anopheles* species, 5.7%. Recent studies demonstrated that *An*. *gambiae* s.l in this area consists entirely of *An*. *arabiensis*, while *An*. *funestus* comprised >95% *An*. *funestus s*.*s*, only <5% being other siblings, e.g. *An*. *rivulorum* and *An*. *leesoni* [19,20,23].

**Table 3:** Number of mosquitoes of different species collected indoors and outdoors using CDC-light traps or miniaturized double net traps (DN-Mini) in the study area°.

Trap type	No. trap nights	No. houses	Mosquito species	No. mosquitoes indoors (%)	No. mosquitoes outdoors (%)	Total
CDC light trap	1,620	90	*Anopheles arabiensis*	7,766 (64.2%)	4,330 (35.8%)	12,096
			*Anopheles funestus*	238 (56.7%)	182 (43.3%)	420
			Other *Anopheles* species	69 (7.1%)	698 (92.9%)	767
			*Mansonia spp*.	1,182 (31.4%)	2,578 (68.6%)	3,760
			*Culex spp*.	92,005 (66.6%)	46,039 (33.4%)	138,044
			Other mosquito species	422 (30.3%)	947 (69.7%)	1,369
DN-Mini trap	320	8	*Anopheles arabiensis*	125 (29.1%)	304 (70.9%)	429
			*Anopheles funestus*	58 (62.4)	35 (37.6%)	93
			Other *Anopheles* species	4 (28.1%)	13 (71.9%)	17
			*Mansonia spp*.	13 (4.4%)	282 (95.6%)	295
			*Culex spp*.	4,744 (61.4%)	2,983 (38.6%)	7,727
			Other mosquito species	3 (26.7%)	8 (73.3%)	11

°An initial assessment of catches using the CDC-light trap catches indoors and outdoors revealed a misrepresentation of the known indoor-outdoor biting proportions of malaria vectors as most recently observed field collections by adult male human volunteers [20]. Therefore, for purposes of assessing the correlations with human activities, only the data from the DN-Mini traps was used.

**Fig 6:**
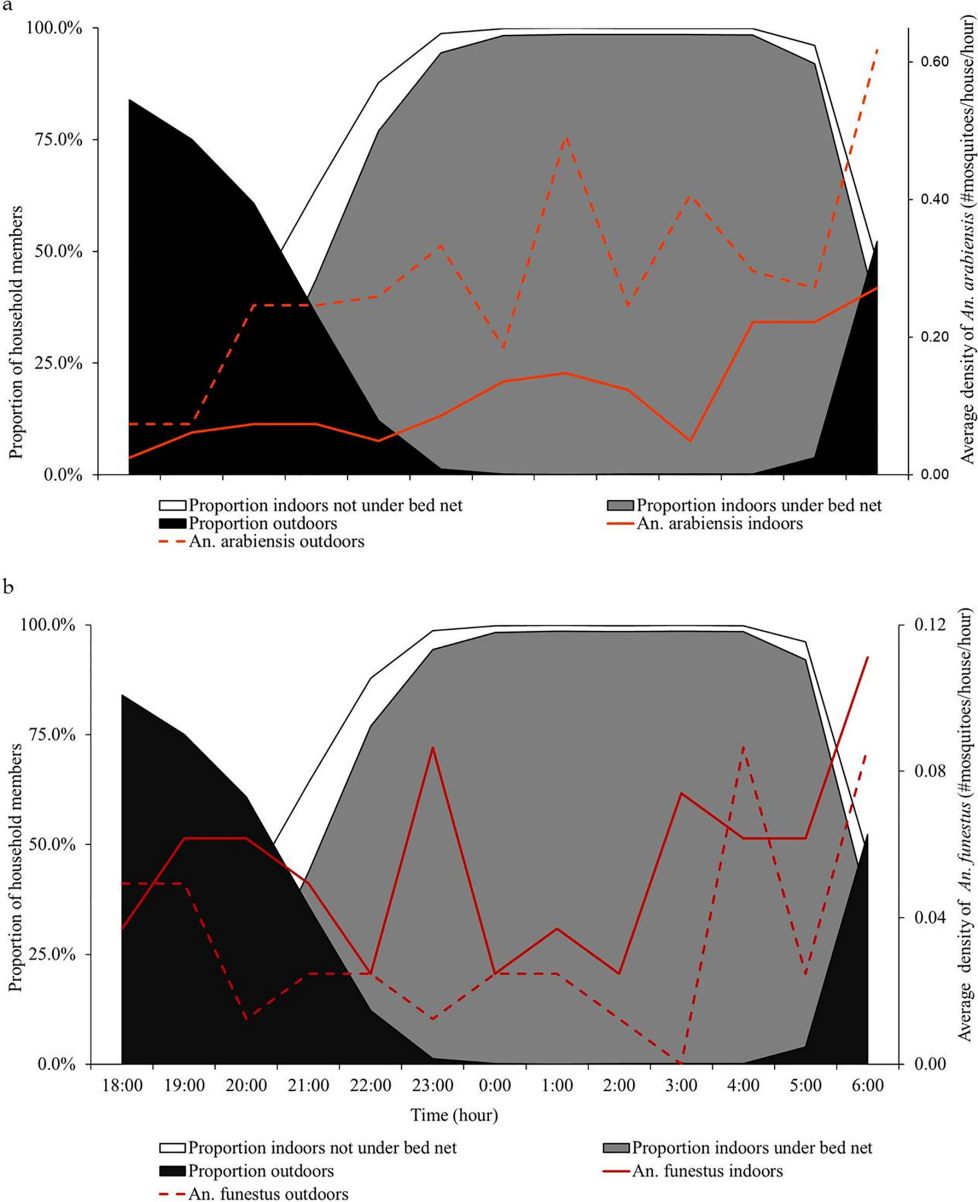
**Illustration of human activity (outdoors, indoors under nets, or indoors outside nets), and biting activity of (a) *An*. *arabiensis* and (b) *An*. *funestus* mosquitoes, measured using miniaturized double net trap (DN-Mini), at different times of night**.

Indoor and outdoor densities of malaria vectors were compared using data from DN-Mini traps (Fig 6). More *An*. *arabiensis* were caught outdoors throughout the night (70.6%) than indoors (29.4%). However, for *An*. *funestus*, catches were higher indoors (63.9%) than outdoors (36.1%). There were no clear peaks in biting time, but indoor and outdoor densities of *An*. *arabiensis* increased even as people moved from outdoors to indoors. For *An*. *funestus*, the biting densities remained generally constant throughout the night, though there were slightly higher rates in early mornings. Additionally, higher densities of *An*.*arabiensis* and *An*.*funestus* were observed in the rural compared to urban settings (Table 4).

**Table 4:** House characteristics and exposure to mosquito bites in the study villages, and estimates of indoor exposure associated with the different vector species.

Species	Characteristics	Indoor			Outdoor		
		Mean ± 2SE	RR (95% CI)	p-value	Mean ± 2SE	RR (95% CI)	p-value
***Anopheles arabiensis***	*Location*						
	Urban	102.53 ± 60.5	1		46.53 ± 29.83	1	
	Rural	177.94 ± 69.89	1.39 (0.64, 3.03)	p = 0.40	103.78 ± 41.34	2.60 (1.30, 5.20)	p < 0.01
	*Window screening*						
	Unscreened	189.96 ± 91.31	1		95.88 ± 53.38	1	
	Screened	120.68 ± 51.28	0.92 (0.43, 1.95)	p = 0.82	77.32 ± 32.32	1.07 (0.54, 2.10)	p = 0.84
	*Electricity*						
	No	176.66 ± 61.64	1		99.05 ± 36.60	1	
	Yes	58.11 ± 50.03	0.28 (0.11, 0.74)	p <0.05	29.89 ± 16.81	0.29 (0.12, 0.69)	p < 0.01
	*Wall type*						
	Brick	192.80 ± 78.18	1		110.27 ± 47.77	1	
	Mud	99.1 ± 53.51	0.48 (0.23, 1.02)	p <0.05	51.10 ± 23.27	0.40 (0.20, 0.77)	p < 0.01
	*Roof type*						
	Metal	114.56 ± 41.46	1		83.00 ± 39.43	1	
	Thatch	241.94 ± 132.34	1.57 (0.67, 3.59)	p = 0.28	94.25 ± 50.12	0.65 (0.31, 1.37)	p = 0.26
***Anopheles funestus***	*Location*						
	Urban	2.00 ± 1.95	1		1.27 ± 1.33	1	
	Rural	5.94 ± 2.12	2.64 (1.11, 6.25)	p <0.05	4.66 ± 1.95	2.77 (1.16, 6.66)	p<0.05
	*Window screening*						
	Unscreened	5.24 ± 1.97	1		3.72 ± 2.11	1	
	Screened	4.28 ± 2.70	1.15 (0.52, 2.53)	p = 0.73	3.56 ± 2.12	1.39 (0.63, 3.09)	p = 0.42
	*Electricity*						
	No	5.61 ± 1.92	1		4.19 ± 1.74	1	
	Yes	0.89 ± 1.15	0.15 (0.04, 0.53)	p < 0.01	1.11 ± 1.32	0.33 (0.10, 1.06)	p = 0.06
	*Wall type*						
	Brick	4.87 ± 1.95	1		3.57 ± 1.91	1	
	Mud	4.60 ± 3.01	0.89 (0.40, 1.95)	p = 0.76	3.75 ± 2.40	1.21 (0.54, 2.68)	p = 0.64
	*Roof type*						
	Metal	4.03 ± 1.91	1		2.76 ± 1.37	1	
	Thatch	6.31 ± 3.19	1.15 (0.49, 2.70)	p = 0.74	5.50 ± 3.50	1.54 (0.65, 3.65)	p = 0.32

### Actual exposure to malaria vectors outdoors, indoors and during bed net use

**Fig 7:**
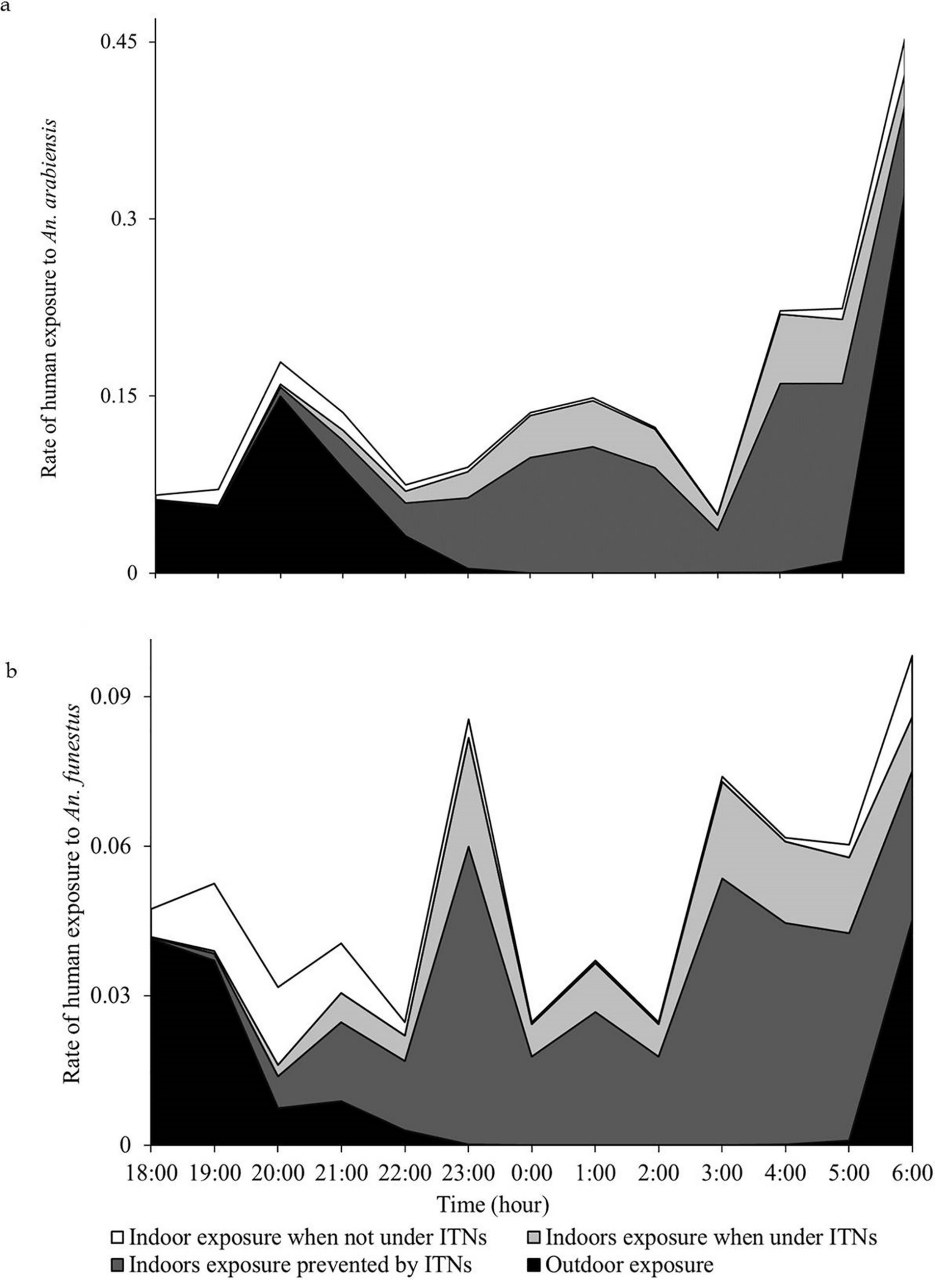
**Hourly exposure to indoor and outdoor mosquito bites and proportions preventable using bed nets: (a) *An*. *arabiensis*; (b) *An*. *funestus***.

Overall estimated proportion of exposure indoors was 63.1% for *An*. *arabiensis* and 78.2% for *An*. *funestus*. Between 18:00 and 21:00h, 78.8% of exposure to *An*. *arabiensis* and 55.1% of exposure to *An*. *funestus* occurred outdoors. However, from 22:00 to 05:00h, nearly all exposure, i.e. 95.5% to *An*. *arabiensis* and 99.0% to *An*. *funestus* occurred indoors. Outdoor exposure increased to 50.7% and 70.9% for *An*. *arabiensis* and *An*. *funestus* respectively from 05:00 to 07:00h (Fig 7).

### Effects of house characteristics on malaria vector biting exposure

Three quarters (75.5%) of the houses did not have electricity. Houses with electricity had significantly lower indoor densities of both *An*. *arabiensis* [RR = 0.34 (0.13–0.92), P<0.05] and *An*. *funestus* [RR = 0.16 (0.05–0.53), P<0.05], and lower outdoor densities of *An*. *funestus* [RR = 0.29 (0.09–0.94), P<0.05] compared to houses with no electricity (Table 4). On the other hand, houses in rural settings had significantly higher outdoor densities of both *An*. *arabiensis* [RR = 2.19 (1.07–4.51), P<0.05] and *An*. *funestus* [RR = 3.22 (01.34–7.76), P<0.05], and higher indoor densities of *An*. *funestus* [RR = 2.82 (1.21–6.61), P<0.05] compared to houses in urban settings (Table 4). No significant difference in indoor or outdoor densities of both species was observed in houses with screened or unscreened windows, or in houses with or without domestic animals. Additionally, no significant difference in mosquito density was observed between houses with mud and brick walls, or between houses with thatch and metal roofs (Table 4).

### People’s perceptions regarding malaria transmission, how it happens, when it happens, its risk factors and historical trends

Thirty of the 42 participants were females and 12 were males, and had an average age of 38.9 years (range: 19–72 years). A majority (32) had primary school education; five had no formal education, four had secondary school education and one had college education. Farming was the most common income generating activity, but there were other professions such as small businesses, motor vehicle repairs, and health care work. Below are popular themes that emerged during the interviews:

*Malaria-transmitting mosquitoes are active late in the night and can transmit from person to person within the same night*: All 42 participants believed that malaria-transmitting mosquitoes are only active between midnight and 2am, a time known in Swahili as ‘*usiku wa manane’*. While mosquitoes were generally believed to be most bothersome in the early evening hours when people were sitting outdoors, these early evening mosquitoes were not believed to transmit malaria. For example, one of the participants said, *“I believe female* Anopheles *mosquitoes come between midnight and 2AM*. *The mosquitoes that come early in the evening do not spread malaria*. *But malaria is a tricky disease*. *You may get it today and get treatment*, *and then three days later you have it again*, *even if you sleep under a net”* (Male, 27 years). The participants reported that in order for mosquitoes to easily pick or transfer the malaria parasite, a person had to be in deep sleep. The participants believed that mosquitoes can pick up parasites from a person with malaria and transfer them to a healthy person in one night: *“I believe that one mosquito can infect a lot of people; if one person in the family has malaria*, *then in one night a mosquito can bite that person*, *take the parasites and pass them to the whole family*. *It is in their nature to spread malaria* …*”* (Female, 45 years).

*Mixed reactions on whether malaria transmission has increased or decreased over the years*: Twenty-two of the 42 participants believed transmission had increased in recent years, primarily because they were hearing a lot about it and were often tested for it: “*I want to say that there is more malaria now*, *because I hear about it more now*. *In the past maybe*, *people got sick a lot*, *but they would just say they have fever*, *or headache*. *And there were very few people around here too*. *Now I hear about malaria everywhere I go*. *People talk about it a lot*. *When someone has a headache or a stomach ache*, *they say it is malaria*. *I hear about malaria everywhere*, *so it must have increased”* (Male, 49 years). Seventeen of the 42 participants believed that malaria had decreased, as they had fewer instances of family members getting sick compared to the past: “*I think there is less malaria now*. *My children are all grown up*, *so I would not even know if they get malaria*. *Now I live with my grandchildren*, *but even with them there are fewer malaria episodes*.*”* (Female, 54 years). Three participants said that it was not easy to know if malaria cases had increased or declined, since the population has grown over the past years: “*In the past there were very few people living in this village*, *so if for example 20 people got malaria*, *that was a lot*. *Now the population in the village has grown so much*, *so 20 people can get malaria and it is not a big deal*, *you do not even hear about it*. *Maybe if 200 people get sick then it can be a big deal*. *So*, *it really depends on the population…”* (Male, 27 years).

*Late night activities increase exposure to mosquitoes and malaria*: All participants believed that activities that keep people out until late hours or those that limited their use of core interventions like ITNs increased risk of malaria transmission. Examples included fishing and farming (mostly in the distant river valley) as well as night-parties and funerals. Participants reported that these activities left people vulnerable to malaria-transmitting mosquitoes despite widespread availability of bed nets as this participant explained: *“Every time I come back home after the farming season*, *I discover that I have malaria*. *It is hard to keep mosquitoes away when in the farms*. *There are just too many*, *and nothing works there*.*”* (Male, 31 years). Another participant said: “*There are just so many mosquitoes in the river*. *Soon as you get out of the river*, *they are all on you*. *If you have repellent then you put it on*, *when you get out of the water”* (Male, 27 years).

Women and children were considered most at risk of malaria. Children were believed to be unable to protect themselves, so they depended on their parents to protect them. Women on the other hand were believed to have increased risk because they tended to sit at one place for a long durations, for example when cooking, and that they tended to wear more “revealing clothing” than men, as this participant explained, *“Children cannot ward off mosquitoes; they are always busy playing or sleeping*, *so it depends on their mothers to protect them*. *And other times*, *because it is hot*, *they wear small clothes*, *so mosquitoes can get them more easily… and women do not wear clothes that cover all the body*.*”* (Female, 20 years).

*Bed nets are still the main malaria prevention tool but they are imperfect*, *yet no alternatives are available*: All 42 participants reported using bed nets every night throughout the year. While outdoors, the participants reported warding off mosquitoes by waving clothes, paper or their hands. Only nine of the 42 participants reported using topical repellent-lotions whenever they could afford it.

A major complaint expressed by the participants was that bed nets did not always prevent mosquitoes from entering and biting occupants, nor did they always kill mosquitoes. The complaint was mostly directed toward the freely-distributed nets, which participants said have large holes: *“Something that bothers me is that sometimes we are told that these mosquito nets have insecticides*, *but that is a big lie*. *My net*, *I was told that it has insecticide*, *but I tell you*, *every day I find mosquitoes inside*, *even when it was new*. *So then I know it is not treated with insecticides”* (Male, 35 years). Another challenge mentioned was lack of easy-to-use and effective methods for preventing bites during night-time activities such as farming, fishing, funerals and other gatherings: *“Nothing works when we are fishing in the river*. *Nets or even the repellent lotions do not work*, *mosquitoes just keep coming*.*” (*Male, 35 years). Another participant said: *“It is difficult to keep mosquitoes away in the farms*. *Most people do not bring their bed nets there because there is no place to tie it*. *So when you bring one everyone wants to sleep in it*. *Sometimes even four people sleep under one-bed net*, *so it does not offer much protection”* (Male, 31 years). Other participants highlighted more specifically the need for personal protection products, such as topical repellents, in places where people congregate at night. *“When I go to a funeral or a party*, *it would be great if I could have a topical repellent*, *but I cannot always afford it*, *then I just risk being bitten”* (Female, 52 years).

## Discussion

Malaria transmission is influenced by biting patterns and infectiousness of different vector species, but also by human activities and behaviours [37]. Indeed, quantitative assessments of malaria transmission risk illustrate that it requires both the hazard (host-seeking, sporozoite-positive *Anopheles*) and exposure (susceptible humans at the right place and time coincident with the biting *Anopheles*) to be present [38]. This study assessed multiple human activities indoors and outdoors, as well as opinions and perceptions of the people, and also examined the biting behaviours of two major malaria vectors, *An*. *arabienssis* and *An*. *funestus* in rural and urban south eastern Tanzania villages. The main objectives were to identify major drivers of persistent malaria transmission exposure and to identify gaps and new opportunities for accelerating vector control beyond the current best tool, i.e. insecticide-treated nets. Overall, because of human behaviour and vector behaviors, human exposure to both *An*. *arabiensis* and *An*. *funestus* was higher indoors than outdoors regardless of the observed feeding preferences of these mosquito species. A majority of people moved indoors after 21:00h, from which point onwards till 05:00h, exposure was prevented by the relatively high bed net use in the area. On the other hand, outdoor exposure was higher than indoor exposure between 18:00h and 21:00h, and again between 05:00h and 07:00h because majority of the household members were mostly outdoors during these hours. Common activities, including cooking, eating and relaxing were conducted outdoors. These activities involved people sitting in one place for extended periods, hence more vulnerable to mosquito bites.

Although a few in-depth interview participants mentioned that they occasionally used topical repellents when outdoors or away from home, none of these were observed during peri-domestic and non-peri-domestic activity observations. The observations, coupled with similar findings from other settings in the country [16,17,39,40] suggest there is little to no protection during outdoor activities at or away from home. While indoor bites can be prevented with ITNs, the outdoor exposure mostly remains untargeted. This observation indicates critical gaps is exposure outdoors in early evenings and in the mornings, even if the overall contribution of outdoor biting is lower than indoors.

Bradley *et al* [41] claims that increased outdoor biting does not influence malaria burden since the highest exposure to mosquito bites still occurs indoors. In this current study however, people were generally protected by ITNs while indoors, hence more attention is needed on periods when people are vulnerable outdoors. The high bed net access and use correlates with the reduced proportion of indoor exposure to mosquito bites for both species, and further stresses the need to develop and improve protection outdoors. While entomological surveillance was not done away from peri-domestic settings, people that were away from peri-domestic settings were more likely to be spending time outdoors, and were more likely to be unprotected from mosquito bites.

There were also mixed views on whether malaria transmission had increased over the recent years. Although, previous studies done in the Kilombero Valley have shown a gradual decline in malaria transmission over the past few decades [21,22,42,43], the most recent school survey indicated that the study area is still classified as meso-to holoendemic, with prevalence rates above 30%[44]. The belief that malaria transmission has increased may be exacerbated by increased marketing of malaria control interventions, and increased availability of these interventions as well as widely available diagnosis and treatment for malaria. It is necessary to ensure that accurate and adequate information is communicated to the public to avoid any confusion that may arise. Moreover, regular parasitological surveys are necessary to accurately understand the real malaria prevalence and guide interventions. As previously observed [23,45], household characteristics had influence on biting risk at the households. Households with electricity had significantly lower densities of both *An*. *arabiensis* and *An*. *funestus*. Likewise, households in urban settings had lower densities of both vector species compared to households in rural settings, confirming associations between urbanization and improved housing with reduced malaria burden [46–49], and further indicating the need to prioritize the lowest income groups when addressing outdoor exposure.

One of the major limitations of this study was entomological data collections. Ifakara Health Institute no-longer allows use of human landing catches (HLC) method for malaria vector sampling. Thus the study initially used CDC-light traps for indoor and outdoor collections. However, results with this method were incongruent to the last comparative surveys by HLC method, which had demonstrated higher densities of *An*. *arabiensis* outdoors than indoors [20,23]. To minimize this bias, additional entomological samplings were done using DN-Mini traps, and the findings were used to compare indoor and outdoor risk of exposure to mosquito bites. This was however done only in four villages rather than all the nine villages where human activity observations had been done. A second limitation was that people could potentially change their behaviours due to presence of an observer [50]. To limit this effect, this study relied on trained household members for the observation. Since the selection of non peri-domestic observation points was done based on local information from household members and leaders, it is possible that there were biases in the observations and possible misrepresentation of the full spectrum of activities happening away from homes. Thirdly, since the IDIs were done during the day at homes, majority (30 of the 42) of the respondents were women, which has likely created a gender bias in the themes. Finally, in the analyses of household characteristics and malaria risk, we observed that certain characteristics such as having house-screening, having electricity in the house and living under metal roofs were strongly associated with lower mosquito densities, and also that houses in rural areas had more mosquitoes than in rural areas. We recognize that there is a likely co-linearity between some of these factors, which we did not assess in this analysis. For example, houses in urban areas are often more likely to be electrified than those in rural areas, and also more likely to be screened and to have metal roofs. As a result, these multiple factors will likely influence malaria risk in the same direction and should not be considered singly.

## Conclusion

ITNs, where properly used, can still prevent most indoor exposures. However, significant risk continues unabated before bedtime, outdoors in peridomestic spaces, at communal gatherings, and during occupations such as farming and fishing. This remaining risk is greater in rural than urban areas and in low-income than high income households. These require scalable approaches to complement ITNs, particularly targeting outdoor-biting. The socio-ecenomic linkages with this persistent exposure also suggests that the complementary interventions, particularly those targeting outdoor exposure should prioritize low-income families. Second, the discordance in community understanding of persistent transmission and its control, and the feedback on imperfections of current approaches should be considered to update malaria-related communication, and improve future interventions.

## Supporting information

S1 File Peri-domestic activity observation sheet. (PDF)

S2 File Household characteristics and mosquito density data. (CSV)

S3 File In-depth interview guide to explore awareness and perceptions of community members regarding malaria transmission. (PDF)

S4 File Consent form for the participant in Fig 3. (PDF)

S5 File Data on human movement. (CSV)

S6 File A summary of activities that keep people away from home. (PDF)
