# Supplementary material for: Linking human behaviours and malaria vector biting risk in south-eastern Tanzania
Source: PLoS One. 2019 Jun 3;14(6):e0217414. doi: 10.1371/journal.pone.0217414 (PMC6546273; doi:10.1371/journal.pone.0217414)
Supplement: S1 File — (PDF) [file pone.0217414.s001.pdf]

Investigating magnitude and drivers of persistent *Plasmodium* infections in east and West Africa

**Household ID**	
**Number of people living in the household**	
**Date of observation**	

Please write number of people doing each of the following activities for every half-hour slot.

**Activities**	**18:00-18:29**						**18:30-18:59**						**19:00-19:29**					
	**Indoors**		**Outdoors**		**Away**		**Indoors**		**Outdoors**		**Away**		**Indoors**		**Outdoors**		**Away**	
	**M**	**F**	**M**	**F**	**M**	**F**	**M**	**F**	**M**	**F**	**M**	**F**	**M**	**F**	**M**	**F**	**M**	**F**
Children under 5 away																		
Other members away																		
Children under 5 playing																		
Other members playing																		
Children under 5 cooking																		
Other members cooking.																		
Children under 5 eating																		
Other members eating																		
Children under 5 telling/listening to stories inside net																		
Children under 5 telling/listening to stories without net																		
Other members telling/listening to stories inside net																		
Other members telling/listening to stories without net																		
Children under 5 walking/passing																		
Other members walking/passing																		
Children under 5 washing dishes																		
Other members washing dishes																		
Children under 5 watching TV																		
Other members watching TV																		
Children under 5 buying/selling																		
Other members buying/selling																		
Children under 5 bathing																		
Other members bathing																		

Activities	18:00-18:29						18:30-18:59						19:00-19:29					
	Indoors		Outdoors		Away		Indoors		Outdoors		Away		Indoors		Outdoors		Away	
	M	F	M	F	M	F	M	F	M	F	M	F	M	F	M	F	M	F
Children under 5 sleeping inside net																		
Children under 5 sleeping without net																		
Other members sleeping inside net																		
Other members sleeping without net																		
Children under 5 putting children to sleep																		
Other members putting children to sleep																		
Children under 5 resting inside net																		
Children under 5 resting without net																		
Other members resting inside net																		
Other members resting without net																		
Children under 5 milking cows/goat/sheep																		
Other members milking cows/goat/sheep																		
Children under 5 washing clothes																		
Other members washing clothes																		
Children under 5 fetching water																		
Other members fetching water																		
Children under 5 braiding hair																		
Other members fetching water																		
Children under 5 reading/writing/studying																		
Other members reading/writing/studying																		
Children under 5 carrying children																		
Other members carrying children																		
Children under 5 being carried																		
**Other activities**																		

Activities	19:30-19:59						20:00-20:29						20:30-20:59					
	Indoors		Outdoors		Away		Indoors		Outdoors		Away		Indoors		Outdoors		Away	
	M	F	M	F	M	F	M	F	M	F	M	F	M	F	M	F	M	F
Children under 5 away																		
Other members away																		
Children under 5 playing																		
Other members playing																		
Children under 5 cooking																		
Other members cooking.																		
Children under 5 eating																		
Other members eating																		
Children under 5 telling/listening to stories inside net																		
Children under 5 telling/listening to stories without net																		
Other members telling/listening to stories inside net																		
Other members telling/listening to stories without net																		
Children under 5 walking/passing																		
Other members walking/passing																		
Children under 5 washing dishes																		
Other members washing dishes																		
Children under 5 watching TV																		
Other members watching TV																		
Children under 5 buying/selling																		
Other members buying/selling																		
Children under 5 bathing																		
Other members bathing																		

Activities	19:30-19:59						20:00-20:29						20:30-20:59					
	Indoors		Outdoors		Away		Indoors		Outdoors		Away		Indoors		Outdoors		Away	
	M	F	M	F	M	F	M	F	M	F	M	F	M	F	M	F	M	F
Children under 5 sleeping inside net																		
Children under 5 sleeping without net																		
Other members sleeping inside net																		
Other members sleeping without net																		
Children under 5 putting children to sleep																		
Other members putting children to sleep																		
Children under 5 resting inside net																		
Children under 5 resting without net																		
Other members resting inside net																		
Other members resting without net																		
Children under 5 milking cows/goat/sheep																		
Other members milking cows/goat/sheep																		
Children under 5 washing clothes																		
Other members washing clothes																		
Children under 5 fetching water																		
Other members fetching water																		
Children under 5 braiding hair																		
Other members fetching water																		
Children under 5 reading/writing/studying																		
Other members reading/writing/studying																		
Children under 5 carrying children																		
Other members carrying children																		
Children under 5 being carried																		
**Other activities**																		

Activities	21:00-21:29						21:30-21:59						22:00-22:29					
	Indoors		Outdoors		Away		Indoors		Outdoors		Away		Indoors		Outdoors		Away	
	M	F	M	F	M	F	M	F	M	F	M	F	M	F	M	F	M	F
Children under 5 away																		
Other members away																		
Children under 5 playing																		
Other members playing																		
Children under 5 cooking																		
Other members cooking.																		
Children under 5 eating																		
Other members eating																		
Children under 5 telling/listening to stories inside net																		
Children under 5 telling/listening to stories without net																		
Other members telling/listening to stories inside net																		
Other members telling/listening to stories without net																		
Children under 5 walking/passing																		
Other members walking/passing																		
Children under 5 washing dishes																		
Other members washing dishes																		
Children under 5 watching TV																		
Other members watching TV																		
Children under 5 buying/selling																		
Other members buying/selling																		
Children under 5 bathing																		
Other members bathing																		

Activities	21:00-21:29						21:30-21:59						22:00-22:29					
	Indoors		Outdoors		Away		Indoors		Outdoors		Away		Indoors		Outdoors		Away	
	M	F	M	F	M	F	M	F	M	F	M	F	M	F	M	F	M	F
Children under 5 sleeping inside net																		
Children under 5 sleeping without net																		
Other members sleeping inside net																		
Other members sleeping without net																		
Children under 5 putting children to sleep																		
Other members putting children to sleep																		
Children under 5 resting inside net																		
Children under 5 resting without net																		
Other members resting inside net																		
Other members resting without net																		
Children under 5 milking cows/goat/sheep																		
Other members milking cows/goat/sheep																		
Children under 5 washing clothes																		
Other members washing clothes																		
Children under 5 fetching water																		
Other members fetching water																		
Children under 5 braiding hair																		
Other members fetching water																		
Children under 5 reading/writing/studying																		
Other members reading/writing/studying																		
Children under 5 carrying children																		
Other members carrying children																		
Children under 5 being carried																		
**Other activities**																		

Activities	22:30-22:59						23:00-23:29						23:30-23:59					
	Indoors		Outdoors		Away		Indoors		Outdoors		Away		Indoors		Outdoors		Away	
	M	F	M	F	M	F	M	F	M	F	M	F	M	F	M	F	M	F
Children under 5 away																		
Other members away																		
Children under 5 playing																		
Other members playing																		
Children under 5 cooking																		
Other members cooking.																		
Children under 5 eating																		
Other members eating																		
Children under 5 telling/listening to stories inside net																		
Children under 5 telling/listening to stories without net																		
Other members telling/listening to stories inside net																		
Other members telling/listening to stories without net																		
Children under 5 walking/passing																		
Other members walking/passing																		
Children under 5 washing dishes																		
Other members washing dishes																		
Children under 5 watching TV																		
Other members watching TV																		
Children under 5 buying/selling																		
Other members buying/selling																		
Children under 5 bathing																		
Other members bathing																		

Activities	22:30-22:59						23:00-23:29						23:30-23:59					
	Indoors		Outdoors		Away		Indoors		Outdoors		Away		Indoors		Outdoors		Away	
	M	F	M	F	M	F	M	F	M	F	M	F	M	F	M	F	M	F
Children under 5 sleeping inside net																		
Children under 5 sleeping without net																		
Other members sleeping inside net																		
Other members sleeping without net																		
Children under 5 putting children to sleep																		
Other members putting children to sleep																		
Children under 5 resting inside net																		
Children under 5 resting without net																		
Other members resting inside net																		
Other members resting without net																		
Children under 5 milking cows/goat/sheep																		
Other members milking cows/goat/sheep																		
Children under 5 washing clothes																		
Other members washing clothes																		
Children under 5 fetching water																		
Other members fetching water																		
Children under 5 braiding hair																		
Other members fetching water																		
Children under 5 reading/writing/studying																		
Other members reading/writing/studying																		
Children under 5 carrying children																		
Other members carrying children																		
Children under 5 being carried																		
**Other activities**																		

Activities	00:00-00:29						00:30-00:59						01:00-01:29					
	Indoors		Outdoors		Away		Indoors		Outdoors		Away		Indoors		Outdoors		Away	
	M	F	M	F	M	F	M	F	M	F	M	F	M	F	M	F	M	F
Children under 5 away																		
Other members away																		
Children under 5 playing																		
Other members playing																		
Children under 5 cooking																		
Other members cooking.																		
Children under 5 eating																		
Other members eating																		
Children under 5 telling/listening to stories inside net																		
Children under 5 telling/listening to stories without net																		
Other members telling/listening to stories inside net																		
Other members telling/listening to stories without net																		
Children under 5 walking/passing																		
Other members walking/passing																		
Children under 5 washing dishes																		
Other members washing dishes																		
Children under 5 watching TV																		
Other members watching TV																		
Children under 5 buying/selling																		
Other members buying/selling																		
Children under 5 bathing																		
Other members bathing																		

Activities	00:00-00:29						00:30-00:59						01:00-01:29					
	Indoors		Outdoors		Away		Indoors		Outdoors		Away		Indoors		Outdoors		Away	
	M	F	M	F	M	F	M	F	M	F	M	F	M	F	M	F	M	F
Children under 5 sleeping inside net																		
Children under 5 sleeping without net																		
Other members sleeping inside net																		
Other members sleeping without net																		
Children under 5 putting children to sleep																		
Other members putting children to sleep																		
Children under 5 resting inside net																		
Children under 5 resting without net																		
Other members resting inside net																		
Other members resting without net																		
Children under 5 milking cows/goat/sheep																		
Other members milking cows/goat/sheep																		
Children under 5 washing clothes																		
Other members washing clothes																		
Children under 5 fetching water																		
Other members fetching water																		
Children under 5 braiding hair																		
Other members fetching water																		
Children under 5 reading/writing/studying																		
Other members reading/writing/studying																		
Children under 5 carrying children																		
Other members carrying children																		
Children under 5 being carried																		
**Other activities**																		

Activities	01:30-01:59						02:00-02:29						02:30-02:59					
	Indoors		Outdoors		Away		Indoors		Outdoors		Away		Indoors		Outdoors		Away	
	M	F	M	F	M	F	M	F	M	F	M	F	M	F	M	F	M	F
Children under 5 away																		
Other members away																		
Children under 5 playing																		
Other members playing																		
Children under 5 cooking																		
Other members cooking.																		
Children under 5 eating																		
Other members eating																		
Children under 5 telling/listening to stories inside net																		
Children under 5 telling/listening to stories without net																		
Other members telling/listening to stories inside net																		
Other members telling/listening to stories without net																		
Children under 5 walking/passing																		
Other members walking/passing																		
Children under 5 washing dishes																		
Other members washing dishes																		
Children under 5 watching TV																		
Other members watching TV																		
Children under 5 buying/selling																		
Other members buying/selling																		
Children under 5 bathing																		
Other members bathing																		

Activities	01:30-01:59						02:00-02:29						02:30-02:59					
	Indoors		Outdoors		Away		Indoors		Outdoors		Away		Indoors		Outdoors		Away	
	M	F	M	F	M	F	M	F	M	F	M	F	M	F	M	F	M	F
Children under 5 sleeping inside net																		
Children under 5 sleeping without net																		
Other members sleeping inside net																		
Other members sleeping without net																		
Children under 5 putting children to sleep																		
Other members putting children to sleep																		
Children under 5 resting inside net																		
Children under 5 resting without net																		
Other members resting inside net																		
Other members resting without net																		
Children under 5 milking cows/goat/sheep																		
Other members milking cows/goat/sheep																		
Children under 5 washing clothes																		
Other members washing clothes																		
Children under 5 fetching water																		
Other members fetching water																		
Children under 5 braiding hair																		
Other members fetching water																		
Children under 5 reading/writing/studying																		
Other members reading/writing/studying																		
Children under 5 carrying children																		
Other members carrying children																		
Children under 5 being carried																		
**Other activities**																		

Activities	03:00-03:29						03:30-03:59						04:00-04:29					
	Indoors		Outdoors		Away		Indoors		Outdoors		Away		Indoors		Outdoors		Away	
	M	F	M	F	M	F	M	F	M	F	M	F	M	F	M	F	M	F
Children under 5 away																		
Other members away																		
Children under 5 playing																		
Other members playing																		
Children under 5 cooking																		
Other members cooking.																		
Children under 5 eating																		
Other members eating																		
Children under 5 telling/listening to stories inside net																		
Children under 5 telling/listening to stories without net																		
Other members telling/listening to stories inside net																		
Other members telling/listening to stories without net																		
Children under 5 walking/passing																		
Other members walking/passing																		
Children under 5 washing dishes																		
Other members washing dishes																		
Children under 5 watching TV																		
Other members watching TV																		
Children under 5 buying/selling																		
Other members buying/selling																		
Children under 5 bathing																		
Other members bathing																		

Activities	03:00-03:29						03:30-03:59						04:00-04:29					
	Indoors		Outdoors		Away		Indoors		Outdoors		Away		Indoors		Outdoors		Away	
	M	F	M	F	M	F	M	F	M	F	M	F	M	F	M	F	M	F
Children under 5 sleeping inside net																		
Children under 5 sleeping without net																		
Other members sleeping inside net																		
Other members sleeping without net																		
Children under 5 putting children to sleep																		
Other members putting children to sleep																		
Children under 5 resting inside net																		
Children under 5 resting without net																		
Other members resting inside net																		
Other members resting without net																		
Children under 5 milking cows/goat/sheep																		
Other members milking cows/goat/sheep																		
Children under 5 washing clothes																		
Other members washing clothes																		
Children under 5 fetching water																		
Other members fetching water																		
Children under 5 braiding hair																		
Other members fetching water																		
Children under 5 reading/writing/studying																		
Other members reading/writing/studying																		
Children under 5 carrying children																		
Other members carrying children																		
Children under 5 being carried																		
**Other activities**																		

Activities	04:30-04:59						05:00-05:29						05:30-05:59					
	Indoors		Outdoors		Away		Indoors		Outdoors		Away		Indoors		Outdoors		Away	
	M	F	M	F	M	F	M	F	M	F	M	F	M	F	M	F	M	F
Children under 5 away																		
Other members away																		
Children under 5 playing																		
Other members playing																		
Children under 5 cooking																		
Other members cooking.																		
Children under 5 eating																		
Other members eating																		
Children under 5 telling/listening to stories inside net																		
Children under 5 telling/listening to stories without net																		
Other members telling/listening to stories inside net																		
Other members telling/listening to stories without net																		
Children under 5 walking/passing																		
Other members walking/passing																		
Children under 5 washing dishes																		
Other members washing dishes																		
Children under 5 watching TV																		
Other members watching TV																		
Children under 5 buying/selling																		
Other members buying/selling																		
Children under 5 bathing																		
Other members bathing																		

Activities	04:30-04:59						05:00-05:29						05:30-05:59					
	Indoors		Outdoors		Away		Indoors		Outdoors		Away		Indoors		Outdoors		Away	
	M	F	M	F	M	F	M	F	M	F	M	F	M	F	M	F	M	F
Children under 5 sleeping inside net																		
Children under 5 sleeping without net																		
Other members sleeping inside net																		
Other members sleeping without net																		
Children under 5 putting children to sleep																		
Other members putting children to sleep																		
Children under 5 resting inside net																		
Children under 5 resting without net																		
Other members resting inside net																		
Other members resting without net																		
Children under 5 milking cows/goat/sheep																		
Other members milking cows/goat/sheep																		
Children under 5 washing clothes																		
Other members washing clothes																		
Children under 5 fetching water																		
Other members fetching water																		
Children under 5 braiding hair																		
Other members fetching water																		
Children under 5 reading/writing/studying																		
Other members reading/writing/studying																		
Children under 5 carrying children																		
Other members carrying children																		
Children under 5 being carried																		
**Other activities**																		

Activities	06:00-06:29						06:30-07:00					
	Indoors		Outdoors		Away		Indoors		Outdoors		Away	
	M	F	M	F	M	F	M	F	M	F	M	F
Children under 5 away												
Other members away												
Children under 5 playing												
Other members playing												
Children under 5 cooking												
Other members cooking.												
Children under 5 eating												
Other members eating												
Children under 5 telling/listening to stories inside net												
Children under 5 telling/listening to stories without net												
Other members telling/listening to stories inside net												
Other members telling/listening to stories without net												
Children under 5 walking/passing												
Other members walking/passing												
Children under 5 washing dishes												
Other members washing dishes												
Children under 5 watching TV												
Other members watching TV												
Children under 5 buying/selling												
Other members buying/selling												
Children under 5 bathing												
Other members bathing												

Activities	00:00-00:29						00:30-00:59					
	Indoors		Outdoors		Away		Indoors		Outdoors		Away	
	M	F	M	F	M	F	M	F	M	F	M	F
Children under 5 sleeping inside net												
Children under 5 sleeping without net												
Other members sleeping inside net												
Other members sleeping without net												
Children under 5 putting children to sleep												
Other members putting children to sleep												
Children under 5 resting inside net												
Children under 5 resting without net												
Other members resting inside net												
Other members resting without net												
Children under 5 milking cows/goat/sheep												
Other members milking cows/goat/sheep												
Children under 5 washing clothes												
Other members washing clothes												
Children under 5 fetching water												
Other members fetching water												
Children under 5 braiding hair												
Other members fetching water												
Children under 5 reading/writing/studying												
Other members reading/writing/studying												
Children under 5 carrying children												
Other members carrying children												
Children under 5 being carried												
**Other activities**												
