# Supplementary material for: Linking human behaviours and malaria vector biting risk in south-eastern Tanzania
Source: PLoS One. 2019 Jun 3;14(6):e0217414. doi: 10.1371/journal.pone.0217414 (PMC6546273; doi:10.1371/journal.pone.0217414)
Supplement: S3 File — (PDF) [file pone.0217414.s003.pdf]

Guide for conducting semi-structured in-depth interviews, to assess views and opinions of community members on residual malaria transmission, mosquito bites and associated determinants, in English and Kiswahili

**Guide for semi-structured interviews: English and Kiswahili version**
Participant ID/**Kitambulisho cha Mshiriki:** ☐☐☐<br><br>Name of interviewer: ---------------------------- Date of interview: ------------------<br>**Jina la mhojaji: ---------------------------- Tarehe ya mahojiano:----------------**<br>District_______________________ Ward_________________________<br>**Wilaya_______________________ Kata_________________________**<br><br>Village: ___________________ Classification;<br>Rural/peri urban/Urban________<br>**Kijiji: ------------------------------- Uainishaji:**<br>**Vijijini/Mji/Jijini______________**<br><br>Demographic information:<br>**Taarifa za kidemografia:**<br><br>Age of the respondent: -------------------------------<br>**Umri wa anayejibu: ---------------------------------**<br><br>Gender (Sex): -----------------------------------<br>**Jinsia:--**<br><br>Education level: --------------------------------<br>**Kiwango cha elimu:---------------------------**<br><br>Marital status of the respondent---<br>**Anaejibu ameoa/ ajaoa:--**

What is your current occupation?____________________

Unajishughulisha na shughuli gani?_________________

General knowledge on malaria and outdoor transmission

Uelewa wako kuhusiana na malaria na maambukizi yanayotokea nje

- Whatdo you know about malaria?
- **Nini kinasababisha malaria?**

- In your opinion, what time do mosquitoes bite the most?

  Kwa maoni yako, Ni muda gani mbu huwa wanang'ata kwa wingi?
  - Probe where do these bites occur

    Dodosa kuhusu muda ambao mbu hung'ata kwa wingi zaidi.
  - Probe on infectivity of the mosquitoes

    Dodosa kuhusu maambukizi yanavyosababishwa na mbu.

- Where parts of your body do mosquitoes bite the most?

  Ni sehemu gani haswa ya mwili mbu wanang'ata?

Knowledge regarding control of malaria Transmission:

Uelewa wako kuhusiana na udhibiti wa maambukizi ya malaria.

- In your opinion, what time do mosquitoes that cause malaria bite the most?

  Kwa maoni yako, Ni muda gani mbu wanaoeneza malaria wanang'ata sana?

- In your opinion, do you think you are at risk of malaria transmission?

  Kwa mtazamo wako, unafikiri unaweza kuambukizwa malaria kirahisi?
  - Probe on the risk of transmission outdoors

    Dodosa kuhusu athari za maambukizi anapokuwa nje ya nyumba.
  - Probe on the risk of transmission indoors

    Dodosa kuhusu athari anapokuwa ndani ya nyumba.

- Probe on the risk of transmission in non-peridomestic places.

  Dodosa kuhusu athari za maambukizi anapokuwa mbali na nyumbani.

- How do you protect yourself from mosquito bites when you are outdoors?

  Unapokuwa nje ya nyumba, huwa unajikingaje dhidi ya mbu?

  - Probe on the type of clothing

    Dodosa kuhusu aina ya nguo wanazovaa

  - Probe on the use of repellents

    Dodosa kuhusu utumiaji wa dawa za kuzuia mbu.

  - Probe on when they are away from home

    Dodosa kuhusu kujikinga anapokuwa mbali na nyumbani.

Information regarding exposure to mosquito bites indoor and outdoor

Ufahamu kuhusiana na ung'atwaji wa mbu ndani nan je ya nyumba

- How many people live in your household?

  Ni watu wangapi wanaishi katika nyumba yako?

- Do you own a bed net?

  Je unamiliki chandarua?

  - Probe on the age of the net – when was it obtained?

    Dodosa umri wa chandarua – amekuwa nayo kwa muda gani?

- How many bed nets do you have? Unamiliki vyandarua vingapi?

  - Probe on type of the net, whether they are treated or untreated

    Dodosa kama chandarua imetiwa dawa au la.

  - Probe on the state of the net, any repairs if found torn

    Dodosa kuhusu hali ya chandarua, kama zinarekebishwa zikipata vitundu

- How many people in your household sleep under one bed net?

  Ni watu wangapi ndani ya nyumba yako wanalala katika chandarua moja?

  - Probe on the sleeping position inside a net

    Dodosa kuhusu nafasi za kulala ndani ya chandarua

- Do you normally sleep under your bed net? Je ni kawaida yako kulala katika chandarua?

  - Probe when they go to bed

    Dodosa ni muda gani kawaida wanaenda kulala

  - Probe do they immediately enter into the bed net

    Dodosa ni muda gani wanaingia ndani ya neti kama wanaingia ndani

- If you have children, what time do they normally go to bed?

  Kama una watoto, Je ni muda gani kawaida wanaenda kulala?

  - Probe on activities they do before sleeping

    Dodosa kuhusu shughuli wanazozifanya kabla ya kulala

  - Probe if they immediately enter into the bed net

    Dodosa kama wanaigia muda huo huo ndani ya neti

- What do you normally do before going to bed at night?

  Huwa unafanya nini kabla ya kwenda kulala?

  - Probe on what they do when they wake up

    Dodosa wanachofanya wakiamka asubuhi

  - Probe on time they wake up and leave the bed

    Dodosa muda gani wanaamka asubuhi

- What non-peridomestic activities take place in your community that you normally attend?

  Kuna shughuli gani zinazotokea kwenye jamii yako ambazo huwa unahudhuria?

  - Probe on when and where exactly these activities occur

    Dodosa kuhusu wakati na mahali shughuli hizi zinatokea

  - Probe on the nature of the activities in general

    Dodosa kuhusu aina ya shughuli zinazotokea kwa ujumla.
