# Supplementary material for: Linking human behaviours and malaria vector biting risk in south-eastern Tanzania
Source: PLoS One. 2019 Jun 3;14(6):e0217414. doi: 10.1371/journal.pone.0217414 (PMC6546273; doi:10.1371/journal.pone.0217414)
Supplement: S6 File — (PDF) [file pone.0217414.s006.pdf]

Linking human behaviours and malaria vector biting risk in Tanzanian communities where insecticide-treated nets are already widely used

A summary of activities that keep people away from home

A total of 32 non peri-domestic communal activities were observed in the nine villages. These included funerals, weddings, football matches, baptisms, and religious activities (e.g. baptism and first communion) and 40th-day memorials, a common celebration to mark the end of mourning period after a death in a family. Other activities included gatherings in bars, movie kiosks and other informal settings. Activities related to funerals and parties were mostly outdoors, and many lasted all night, while activities associated with bars, movies and football kiosks were both indoors and outdoors, and most lasted until midnight.

Table 2: Proportion of participants of non-peri-domestic activities observed in Kilombero and Ulanga districts

Activity category	# of events observed	# of people observed	Observation time	Males indoors	Males outdoors	Females indoors	Females outdoors
Movies, football matches and bars	13	1,945	18:00-00:00	54.2% (n = 708)	45.8% (n = 598)	53.7% (n = 343)	46.2% (n = 296)
Funerals and 40th-day memorial	11	16,805	18:00 -07:00	6.1% (n = 486)	93.9% (n = 7,463)	12.3% (n = 1,091)	87.7% (n = 7,765)
Weddings and other religious ceremonies	8	7,884	18:00-07:00	7.8% (n = 356)	92.2% (n = 4,208)	14.3% (n = 474)	85.7% (n = 2,846)
